# Gender disparity in minimum dietary diversity failure among currently breastfed children aged 6–23 months in Bangladesh: evidence from Bangladesh Multiple Indicator Cluster Survey, 2019

**DOI:** 10.1017/jns.2023.89

**Published:** 2023-11-03

**Authors:** Md. Ismail Hossain, Samia Kabir, Faozia Afia Zinia

**Affiliations:** 1 Department of Statistics, Jagannath University, Dhaka 1100, Bangladesh; 2 Department of Mathematics and Natural Sciences, BRAC University, Dhaka 1212, Bangladesh

**Keywords:** Child health, Dietary diversity, Minimum dietary diversity failure, Nutrition

## Abstract

Research on children's dietary diversity plays a crucial role in designing effective health interventions. Thus, this study aimed to identify the factors contributing to minimum dietary diversity failure (MDDF) among male and female children aged 6–23 months in Bangladesh. The data for this study was obtained from the Bangladesh Multiple Indicator Cluster Survey, 2019, which included children currently breastfed within a specific age range. Multivariable binary logistic regression was employed to assess the strength and significance of the association. The findings revealed that approximately 59⋅4 % of children in Bangladesh experienced MDDF, with 57⋅8 % of male children and 61 % of female children affected. Proportion test uncovered a significant gender disparity (*χ*^2^=6⋅58, *P*-value = 0⋅01) among children aged 6–23 months. However, the multivariable binary logistic regression analysis revealed that both male and female children shared common risk factors for MDDF, which included child age, maternal educational status, wealth status, number of antenatal care visits, and division. In our study, we observed varied spatial patterns in minimal dietary diversity. Sherpur, Netrokona, Sunamganj, and Sylhet districts showed the highest failure rates. Notably, all are flood-affected areas, impacting food availability and diversity. For targeted regional development programmes, district mapping results may offer valuable insights to policymakers, especially in areas with a high prevalence of dietary diversity failure. By understanding these risk factors, policymakers and stakeholders can implement targeted strategies to improve dietary diversity among children, promoting better health and well-being for the young population in Bangladesh.

## Introduction

Under-five malnutrition in Bangladesh has decreased over time, but it is still far from meeting the Sustainable Development Goals (SDGs).^(1)^ One reason for this malnutrition rate is a lack of adequate dietary diversity among children aged 6–23 months.^(2,3)^ For the proper development of a child before their fifth birthday, the World Health Organization (WHO) developed an indicator named ‘Minimum Dietary Diversity (MDD)’ for feeding children aged 6–23 months, which is defined as the consumption of five or more food items out of eight food groups.^(4)^ These eight food groups are: (a) breast milk, (b) grains, roots, and tubers, (c) legumes and nuts, (d) dairy products, (e) flesh food, (f) eggs, (g) vitamin A-rich fruits and vegetables, and (h) other fruits and vegetables. These foods meet a child's daily nutritional needs. If someone received fewer than five of these eight food groups, they were classified as having a minimal dietary diversity deficiency or minimum dietary diversity failure (MDDF).^(3)^

It is a matter of concern that few children are receiving a nutritionally adequate diet. Globally, only 29 % of infants and young children aged 6–23 months met the criteria of dietary diversity.^(5)^ As a result, 149⋅2 million children under the age of five were stunted, 45⋅4 million were wasted, and 38⋅9 million were overweight, which led to a great risk of child death.^(6)^ The past investigations mentioned that less than one quarter of children in Ethiopia practice a minimum level of dietary diversity.^(7,8)^ This percentage varies in South Asian countries. For example, 15 % of under-five children received the MDD in India,^(9)^ 47 % in Nepal,^(10)^ 21 % in Pakistan,^(11)^ and so on. According to a 2014 estimate in Bangladesh, only 28⋅8 % of children aged 6–23 months meet the minimum of requirements dietary diversity, and one-third of children under-five do not develop before their fifth birthday, which is not enough to ensure the future of a country's children.^(12,13)^ Since nutritional status is perhaps the best indicator of children's well-being,^(14)^ it is therefore important to work towards achieving MDD in the case of Bangladesh.

The reasons for failure in achieving adequate dietary diversity among children aged 6–23 months are numerous and complex. Several studies have been conducted to investigate the determinants of MDD among children in Bangladesh. These studies have highlighted various factors that play a crucial role in determining the MDD, including the residential area,^(12)^ age and sex of the children,^(15)^ education status of mothers/caregivers,^(16)^ wealth index,^(16)^ media access,^(12)^ take health facilities during pregnancy and delivery,^(12)^ and working status.^(15)^ However, despite these efforts, there is still a lack of clear evidence regarding the high prevalence areas of minimal dietary diversity failure, particularly when considering variations between genders. Cultural norms may lead to different food choices for boys and girls, impacting their access to diverse and nutritious diets. The specific risk factors affecting male and female children remain largely undisclosed. To address these gaps in knowledge, the present study aims to comprehensively explore the overall spatial patterns associated with minimal dietary diversity and identify the risk factors influencing dietary inadequacy among boys and girls aged 6–23 months in Bangladesh. By shedding light on gender-specific variations, this research will contribute to a more nuanced understanding of the factors influencing dietary diversity in young children and pave the way for targeted interventions to improve nutrition and overall health outcomes.

## Materials and methods

### Data source

This study utilised the data and variables from the Bangladesh Multiple Indicator Cluster Survey (MICS), 2019, which is a nationally representative cross-sectional survey. The Bangladesh MICS, 2019 was administered by the Bangladesh Bureau of Statistics (BBS) and funded by UNICEF in Bangladesh.

### Sample design and sample size

The Bangladesh MICS, 2019 used a two-stage stratified cluster sampling design, where 3220 enumeration areas were selected in the first stage, and subsequently selected 20 households from each enumeration area in the second stage. The survey covered a total of 64 400 households, and 24 686 mothers/caregivers with children under-five were eligible for the interview. For this study, data were specifically limited to children aged 6–23 months who were currently breastfeeding. Based on this criterion, the weighted sample size for this study was 6092 mothers/caregivers with children aged 6–23 months (comprising 3158 male children and 2934 female children).

### Dependent variable

The MDD score for children aged 6–23 months is defined as the proportion of children aged 6–23 months who consumed foods from at least five of the eight food groups within 24 h.^(17)^ These eight food groups are:

1. breast milk
2. grains, roots, and tubers
3. legumes and nuts
4. dairy products
5. flesh food
6. eggs
7. vitamin A-rich fruits and vegetables
8. other fruits and vegetables

Children who received less than five food groups are categorised as having MDDF, which was the main outcome variable in this study. For pointing out food received for each child *i*, aged of 6–23 months old in Bangladesh for the food group *r*,1
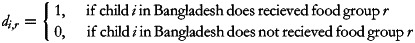


Then, to analyse the MDDF compliance for each child *i*, aged 6–23 months, in Bangladesh, we assess whether they received fewer than five food groups out of eight food groups.

That is,2
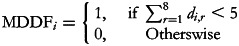


### Independent variables

After the literature review, various socio-demographic and economic variables were included as independent/explanatory characteristics of this study, such as children's age group (in months) (6–14 months, 15–23 months), child sex (Male, Female), maternal education (No/Primary, Secondary, Higher+), wealth status (Poor, Middle, Rich), mass media access (Yes, No), institutional delivery (Yes, No), antenatal care (ANC) visit (None, 1–3, 4+), residence (Urban, Rural), and division (Barisal, Chattogram, Dhaka, Khulna, Mymensingh, Rajshahi, Rangpur, Sylhet).

### Analytical procedure

A simple descriptive analysis, bivariate analysis, and multivariate analysis were conducted in this study. Descriptive analysis describes the percentage distribution of the variables. In bivariate analysis, this study examines the association between MDDF among currently breastfed children aged 6–23 months and selected independent variables. In this case, the Chi-square test statistic is performed, which can be defined as3
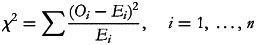
where *O*_*i*_ and *E*_*i*_ are the observed and expected frequency, respectively. The *χ*^2^ statistic asymptotically follows the *χ*^2^ distribution with the degrees of freedom (*a* − 1) × (*b* − 1), where *a* is the number of categories for the independent variable and *b* is the number of categories for the dependent variable.

In a multivariate set-up, the effect of an independent variable on the MDDF among currently breastfed children aged 6–23 months can be determined using the binary logistic regression model. Let *Y*_*i*_ denote the binary dependent variable for the *i*th observation, where
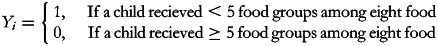


*X*_*i*1_, …, *X*_*ip*_ be a set of independent variables which can be quantitative or indicator variables referring to the level of categorical variables. Since *M*_*i*_ is a binary variable, it has a Bernoulli distribution with parameter *π*_*i*_. The dependent of the probability of success on independent variables is assumed to be respectively as 4
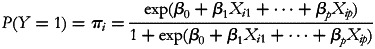


The above relation also can be expressed as5
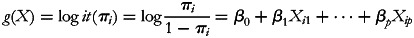


The likelihood is maximised by finding estimates of the parameters that are most likely to give us the data. The maximum likelihood estimator (MLE) of *β*_0_ and *β*_1_ can be obtained by maximising:6
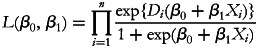


The odds ratio with a 95 % confidence interval was usually used to explain predictor variables’ impact.

### Software

Data wrangling was conducted using Statistical Package for the Social Sciences (SPSS) version 25, while both analysis and geographical mapping were performed using R-programming version 4.0.0.

### Ethical statement

The study utilised publicly available data from the MICS (https://mics.unicef.org/) and downloaded the shape file from the Humanitarian data exchange (https://data.humdata.org/), which was accessible to everyone. Hence, no additional ethical approval was required for this investigation.

## Results

### Geographical conception of MDDF in Bangladesh

**Fig. 1.**
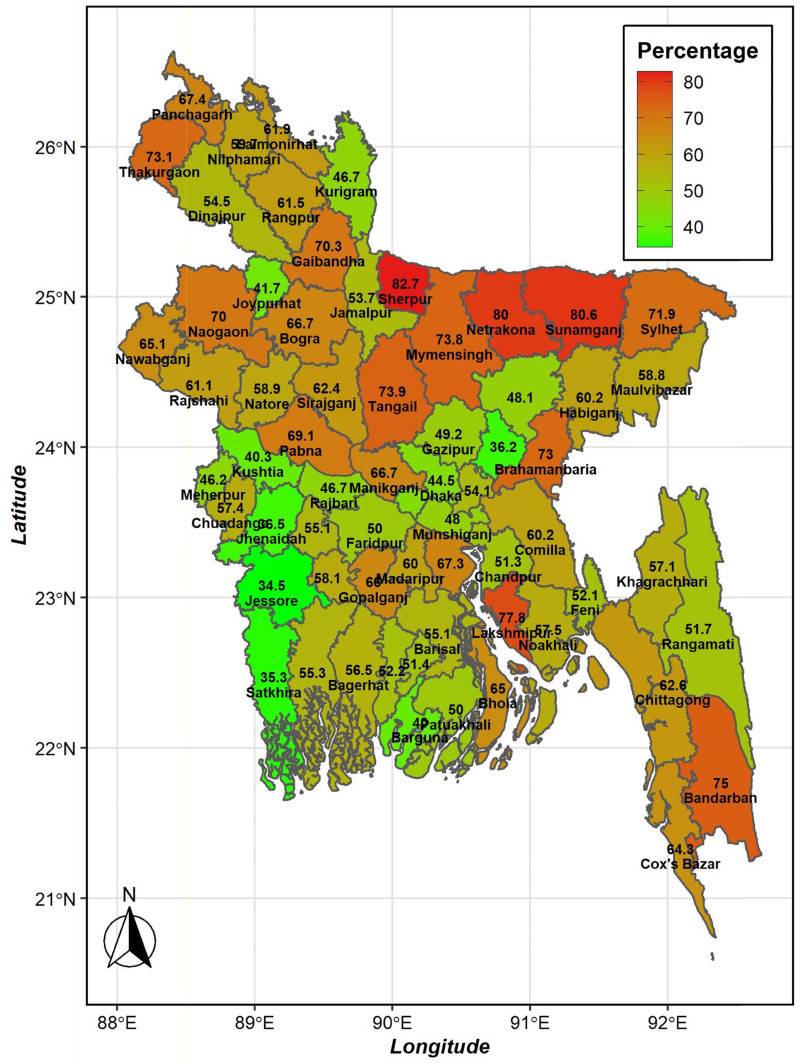
The proportion of minimum dietary diversity failure found for the 64 districts of Bangladesh from the Multiple Indicator Cluster Survey, 2019.

The map presented in Fig. 1 illustrates the percentage of children experiencing MDDF in Bangladesh, categorised by district. The legend represents a gradient from high percentage (red colour) to low percentage (green colour) of MDDF in children aged 6–23 months.

### Prevalence of MDDF in Bangladesh

**Fig. 2.**
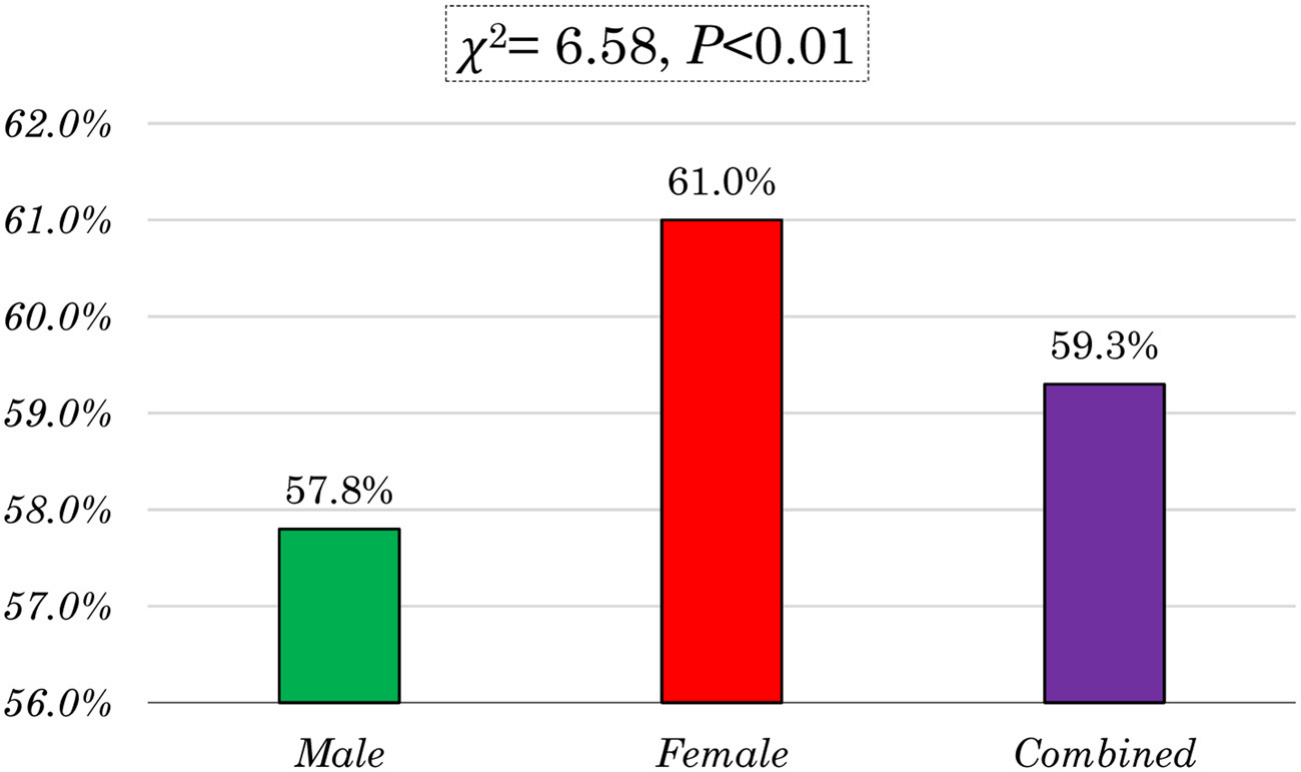
Gender differences of minimum dietary diversity failure in Bangladesh.

Fig. 2 presents the gender differences in the prevalence of MDDF among 6–23-month-old children who were currently breastfeeding in Bangladesh. The figure reveals that 59⋅3 % of the children in this age group received less than five food groups. Specifically, 57⋅8 % of these children were male, while 61 % were female. The bar chart clearly demonstrates that the prevalence of MDD among females aged 6–23 was higher compared to males. Furthermore, the proportion test shows a significant difference between male and female children (*χ*^2^ = 6⋅58, *P* < 0⋅01).

### Frequency of consumed food groups

**Fig. 3.**
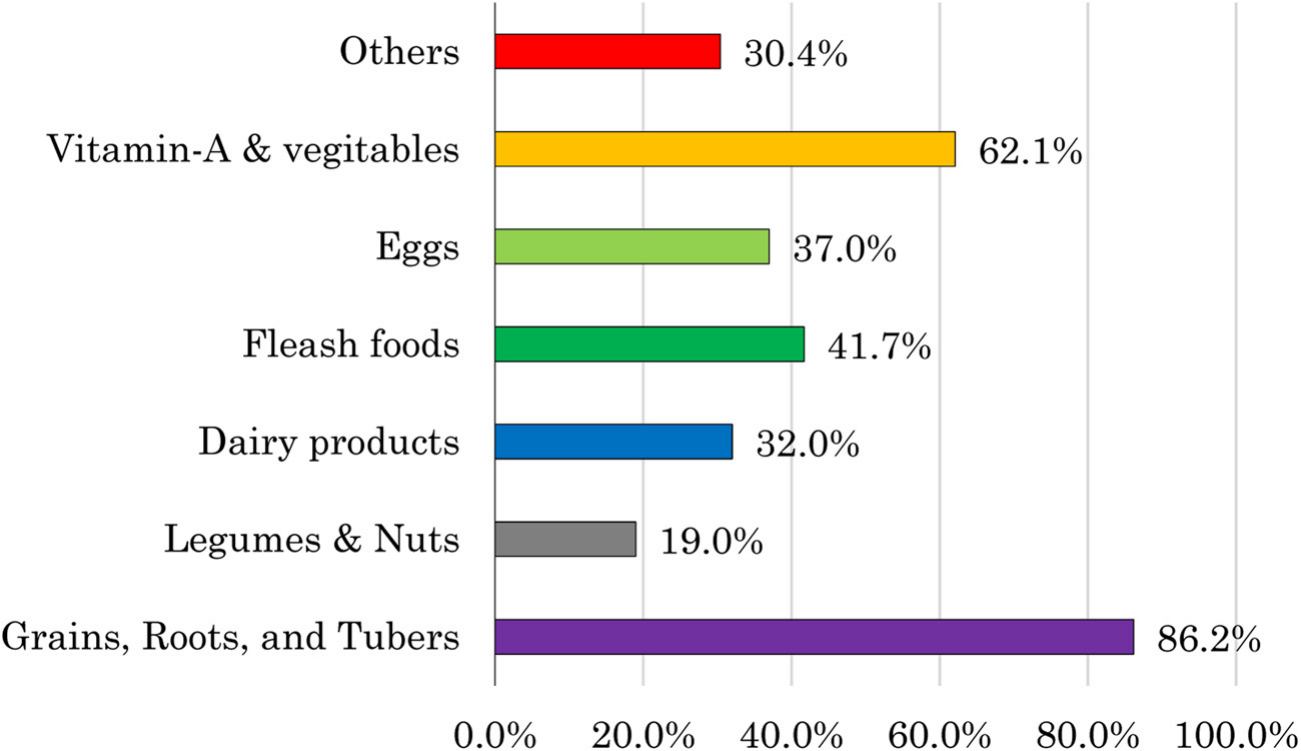
Food groups consumed among 6–23 months children in the last 24 h.

Fig. 3 illustrates the dietary consumption patterns of children within 24 h preceding the survey. Grains, tubers, and roots were the most commonly consumed food group, according to 86⋅2 % of the children's diets. In contrast, the consumption of legumes and nuts was relatively low, with only 19 % of children having included them in their diet.

### Association of socio-demographic and economic factors on MDDF

Table 1 presents the background characteristics of mothers/caretakers and their children included in the study. The largest proportion of mothers resided in Dhaka (23 %) and lived in rural areas (79⋅3 %) of Bangladesh. Nearly half of the respondents had male children (51⋅8 %) and children aged between 6 and 14 months (51⋅6 %). Approximately 41 % of the participants belonged to poor households. Exactly half of the respondents (50 %) had attained a secondary education level. Around two-thirds of the respondents had access to media (60 %), and 45⋅5 % had received 1–3 ANC visits during their pregnancy.

**Table 1.** Exploring the relationship between minimum dietary diversity failure and selected variables (stratified by gender of child)

Variables	Frequency (%)	Minimum dietary diversity failure			
		Male (%)	*χ*^2^-value (*P*-value)	Female (%)	*χ*^2^-value (*P*-value)
Child sex					
Male	3158 (51⋅8)	−	−	−	−
Female	2934 (48⋅2)	−	−	−	−
Child age (in months)					
6–14	3145 (51⋅6)	67⋅3	129⋅30 (<0⋅001)	69⋅5	92⋅40 (<0⋅001)
15–23	2948 (48⋅4)	47⋅3		52⋅2	
Maternal education					
No/Primary	1974 (32⋅4)	68⋅9	104⋅53 (<0⋅001)	71⋅4	116⋅83 (<0⋅001)
Secondary	3047 (50⋅0)	56⋅3		60⋅2	
Higher+	1072 (17⋅6)	42⋅8		42⋅5	
Wealth status					
Poor	2503 (41⋅1)	67⋅4	99⋅10 (<0⋅001)	71⋅8	131⋅39 (<0⋅001)
Middle	1174 (19⋅3)	58⋅8		61⋅8	
Rich	2416 (39⋅7)	47⋅9		48⋅8	
Mass media access					
Yes	3653 (60⋅0)	52⋅4	57⋅81 (<0⋅001)	56⋅7	33⋅75 (<0⋅001)
No	2440 (40⋅0)	66⋅0		67⋅4	
Institutional delivery					
Yes	3149 (52⋅4)	53⋅5	28⋅64 (<0⋅001)	54⋅5	54⋅73 (<0⋅001)
No	2861 (47⋅6)	63⋅0		67⋅9	
ANC visit					
None	1132 (18⋅6)	68⋅1	62⋅44 (<0⋅001)	72⋅6	54⋅97 (<0⋅001)
1–3	2774 (45⋅5)	60⋅3		61⋅9	
4+	2187 (35⋅9)	49⋅3		53⋅7	
Residence					
Urban	1262 (20⋅7)	48⋅8	26⋅49 (<0⋅001)	54⋅1	15⋅77 (<0⋅001)
Rural	4830 (79⋅3)	60⋅0		62⋅9	
Division					
Barisal	342 (5⋅6)	49⋅7	91⋅32 (<0⋅001)	57⋅1	72⋅84 (<0⋅001)
Chattogram	1298 (21⋅3)	61⋅0		64⋅1	
Dhaka	1398 (22⋅9)	52⋅1		52⋅9	
Khulna	656 (10⋅8)	42⋅6		48⋅9	
Mymensingh	476 (7⋅8)	70⋅6		75⋅1	
Rajshahi	751 (12⋅3)	65⋅3		63⋅1	
Rangpur	684 (11⋅2)	56⋅2		66⋅4	
Sylhet	488 (8⋅0)	70⋅3		69⋅5	

Table 1 also presents the relationship between socio-demographic and economic characteristics and the MDDF among 6–23 months male and female children in Bangladesh. Our analysis revealed significant relationships (*P* < 0⋅001) between various variables, including child age (in months), maternal educational status, mass media exposure, wealth status, institutional delivery, antenatal visits, residence, and division of both male and female children in Bangladesh.

Children aged 6–14 months exhibited a higher rate of MDDF, with 67⋅3 % for males and 69⋅5 % for females, compared to those aged 15–23 months, where the rates were 47⋅3 % for males and 52⋅2 % for females. Notably, maternal education showed a significant association with MDDF. Children born to mothers with no or primary education experienced higher failure rates, reaching 68⋅9 % for males and 71⋅4 % for females.

Furthermore, wealth status played a crucial role in MDDF, as children from poor households had elevated failure rates, namely 67⋅4 % for males and 71⋅8 % for females. Access to mass media also exhibited a significant association with failure rates, as children from households without mass media access demonstrated higher rates of failure, reaching 66⋅0 % for males and 67⋅4 % for females.

The mode of delivery emerged as another factor, with institutional delivery associated with lower failure rates (53⋅5 % for males and 54⋅5 % for females). Moreover, children whose mothers had no ANC visits exhibited higher rates of failure, reaching 68⋅1 % for males and 72⋅6 % for females.

Residence played a vital role, as children residing in rural areas displayed higher failure rates compared to their urban counterparts, with rates of 60⋅0 % for males and 62⋅9 % for females. Division also demonstrated a significant association, with considerable variation between males and females across different divisions in terms of failure rates.

These findings underscore the importance of considering factors such as age, maternal education, wealth status, mass media access, delivery mode, ANC visits, residence, and division when addressing MDDF. By targeting these factors, interventions can be tailored to reduce the prevalence of malnutrition and improve dietary diversity among children in Bangladesh.

### Factors associated with MDD among male and female children

Table 2 presents the outcomes of a binary logistic regression analysis exploring the influence of MDDF among male and female children aged 6–23 months based on various background characteristics.

**Table 2.** Binary logistic regression analysis showing the effect of minimum dietary diversity failure among male and female children (6–23 months) by background characteristics

Variables	Male	Female
	OR (95 % CI)	OR (95 % CI)
Child age (in months)		
6–14 (Ref.)	1	1
15–23	0⋅43 (0⋅37–0⋅50)***	0⋅45 (0⋅38–0⋅52)***
Maternal education		
No/Primary (Ref.)	1	1
Secondary	0⋅78 (0⋅65–0⋅94)**	0⋅82 (0⋅67–0⋅99)*
Higher+	0⋅56 (0⋅43–0⋅72)***	0⋅45 (0⋅35–0⋅59)***
Wealth status		
Poor (Ref.)	1	1
Middle	0⋅77 (0⋅62–0⋅95)*	0⋅84 (0⋅67–1⋅05)
Rich	0⋅62 (0⋅49–0⋅77)***	0⋅58 (0⋅46–0⋅73)***
Mass media access		
Yes (Ref.)	1	1
No	1⋅25 (1⋅05–1⋅49)*	1⋅07 (0⋅89–1⋅29)
Institutional delivery		
Yes (Ref.)	1	1
No	0⋅90 (0⋅75–1⋅07)	1⋅21 (1⋅01–1⋅44)*
ANC visit		
None (Ref.)	1	1
1–3	0⋅86 (0⋅69–1⋅08)	0⋅75 (0⋅59–0⋅95)*
4+	0⋅67 (0⋅52–0⋅86)**	0⋅74 (0⋅57–0⋅96)*
Residence		
Urban (Ref.)	1	1
Rural	1⋅01 (0⋅82–1⋅24)	0⋅89 (0⋅72–1⋅10)
Division		
Barisal (Ref.)	1	1
Chattogram	2⋅05 (1⋅50–2⋅82)***	1⋅65 (1⋅21–2⋅23)**
Dhaka	1⋅52 (1⋅11–2⋅09)**	1⋅22 (0⋅89–1⋅67)
Khulna	1⋅19 (0⋅85–1⋅66)	1⋅05 (0⋅77–1⋅45)
Mymensingh	2⋅76 (1⋅82–4⋅21)***	2⋅21 (1⋅44–3⋅39)***
Rajshahi	2⋅29 (1⋅61–3⋅26)***	1⋅58 (1⋅12–2⋅23)**
Rangpur	1⋅62 (1⋅16–2⋅25)**	1⋅68 (1⋅20–2⋅35)**
Sylhet	2⋅55 (1⋅74–3⋅74)***	1⋅67 (1⋅14–2⋅45)**

Statistical Significance: * *P* < 0.05, ** *P* < 0.01, *** *P* < 0.001.

Table 2 reveals that children aged 15–23 months exhibit significantly lower odds of experiencing MDDF in comparison to their younger counterparts (aged 6–14 months) for both male (OR = 0⋅43) and female (OR = 0⋅45) children.

Furthermore, maternal education plays a noteworthy role in MDD outcomes. Male children whose mothers have attained secondary education demonstrate reduced odds (OR = 0⋅78, 95 % CI 0⋅65–0⋅94) of MDDF in contrast to those with no or primary education. Moreover, the effect is even more pronounced among children with mothers having higher educational attainment (OR = 0⋅56, 95 % CI 0⋅43–0⋅72). Similarly, female children also benefit from their mother's education, with those whose mothers have secondary education exhibiting lower odds (OR = 0⋅82, 95 % CI 0⋅67–0⋅99) of MDDF, and even lower odds for those with higher educated mothers (OR = 0⋅45, 95 % CI 0⋅35–0⋅59).

Economic status, as measured by wealth status, is found to be associated with dietary diversity outcomes. Male children from middle households have reduced odds (OR = 0⋅77, 95 % CI 0⋅62–0⋅95) of MDDF compared to those from poor households. The odds are even lower for male children from rich households (OR = 0⋅62, 95 % CI 0⋅49–0⋅77). Among female children, similar patterns emerge, with those from middle households exhibiting decreased odds (OR = 0⋅84, 95 % CI 0⋅67–1⋅05) of MDDF and those from rich households having even lower odds (OR = 0⋅58, 95 % CI 0⋅46–0⋅73). For male children, those without mass media access have 25 % higher odds (OR = 1⋅25, 95 % CI 1⋅05–1⋅49) of MDDF compared to those with access. However, this association was not statistically significant for female children.

Regarding healthcare factors, institutional delivery has no significant association with MDDF among male children. However, for female children, those born outside of institutional delivery have 21 % higher odds (OR = 1⋅21, 95 % CI 1⋅01–1⋅44) of experiencing MDDF. Likewise, the number of ANC visits by mothers appears to be linked to MDDF outcomes for female children. Children whose mothers had 1–3 or 4+ ANC visits exhibit lower odds of MDDF in comparison to those whose mothers had no ANC visits. For instance, those whose mothers had 1–3 ANC visits exhibit lower odds (OR = 0⋅75, 95 % CI 0⋅59–0⋅95) of MDDF, and the odds are even lower for those with 4+ ANC visits (OR = 0⋅74, 95 % CI 0⋅57–0⋅96).

Residence, whether urban or rural, does not seem to have a significant effect on dietary diversity outcomes for both male and female children, significant variations in odds of MDDF are observed across different divisions for both male and female children.

## Discussion

The main objective of this study was to identify risk factors associated with the failure of minimal dietary diversity among boys and girls aged 6–23 months in Bangladesh. Our findings emphasise the urgent need to address the concerning failure rate of achieving MDD. However, approximately two-thirds of 6–23-month-old children do not meet the MDD score in Bangladesh, which was approximately equal to the prevalence observed in Nepal.^(18)^

Through bivariate and multivariate analyses, we identified several significant factors associated with inadequate dietary diversity among children. From bivariate analysis, it was found that child age, maternal education, wealth status, access to media, institutional delivery, prenatal care, place of residence, and divisions were significantly associated with inadequate dietary diversity among children. Subsequently, we used the binary logistic regression model to estimate the adjusted effects of these selected covariates.

The present study's finding revealed that the MDDF rate for female children was 3⋅2 % higher than that of male children. The proportion test indicates there was a significant difference between male and female children aged between 6 and 23 months in Bangladesh. This study was consistent with studies conducted in Ethiopia.^(19,20)^ To gain better understanding of this difference, it is crucial to consider various socio-demographic and economic factors that may contribute to the observed disparity.

The results from binary logistic regression indicate that children aged 15–23 months are less likely to experience a lack of minimal dietary diversity compared to children aged 6–14 months. As children grow older, the risk of not reaching the MDD decreases significantly, as they need a more diverse range of nutrients to support their physical and cognitive development, and failure to meet this changing nutritional need could result in a higher risk of dietary diversity issues. This finding aligns closely with the outcomes of independent studies conducted in Bangladesh and Ethiopia.^(15,21)^

Maternal education is an important indicator influencing children's health and well-being.^(22)^ This study revealed a significant negative association between maternal education and the failure of minimal dietary diversity in both male and female children in Bangladesh. Children with mothers having secondary or higher education faced approximately 18–55 % lower risk of MDDF compared to those born to illiterate mothers. These findings align with another prior study.^(12)^ It is plausible that better-educated mothers tend to have a greater understanding of their children's health needs.^(23)^

There was a notable association between wealth status and inadequate dietary diversity in children. The prevalence of insufficient minimal dietary diversity was relatively lower among children in the rich category of the wealth index compared to their counterparts. However, it is essential to highlight that another prior study has demonstrated that children from poor families face a significantly higher risk of MDDF.^(24)^ Hence, policymakers should take initiatives to not only increase education levels but also raise awareness of child health among economically disadvantaged populations.

Based on the findings of this study, media usage emerged as one of the most important and significant predictors of children's health improvement in Bangladesh, applicable to both boys and girls. The mechanism behind this association can be attributed to the role of media in disseminating health-related information and influencing behaviour. In Bangladesh, media, including television, radio, and digital platforms, often serve as vital channels for health education, including proper child nutrition, breastfeeding practices, and awareness campaigns. Children whose mothers had no access to any type of media were more susceptible to MDDF, consistent with another study conducted in Bangladesh.^(12)^

Mothers who received ANC during pregnancy were less likely to remain in the worst childhood dietary diversity status than mothers who did not use the service. Approximately the same outcome was found in another study conducted in Ethiopia.^(20,25)^ Obviously, ANC plays a crucial role in children's nutrition status and overall health outcomes.^(26)^ For instance, ANC includes nutrition counselling, supplementations, education on breastfeeding, and so on.^(27)^ Moreover, the place of birth had a significant impact on the failure of MDD among children, indicating that mothers who did not have institutional deliveries were at higher risk of MDDF in Bangladeshi children. This result is consistent with several previous studies.^(19)^ Both findings suggest that maternal access to health facilities should be ensured during pregnancy to reduce the failure rate in minimal dietary diversity among children. Additionally, the division also showed a significant impact in this study, revealing that dietary diversity failure was significantly higher among children in all regions of Bangladesh.

This study includes an analysis of dietary diversity failure prevalence across various districts of Bangladesh, identifying districts with high rates, including Sherpur, Netrokona, Sunamganj, and Sylhet. Notably, all these districts are flood-affected areas, which probably contributes to the high prevalence. Floods can disrupt food availability and diversity, leading to rapid changes in dietary patterns within these regions.^(28)^

### Strengths and limitations of the study

The strengths of this study lie in its use of nationally representative data, which provides robustness and applicability to the entire population of interest in Bangladesh. Additionally, the study's focus on gender differences offers valuable insights into potential variations in MDDF between male and female children, contributing to a comprehensive understanding of gender-related factors influencing the outcome. Moreover, the study's calculation of prevalence based on district allows for a detailed assessment of geographical disparities in MDDF, enabling targeted interventions and resources allocation in high prevalence districts like Sherpur, Netrokona, Sunamganj, and Sylhet.

While our study provides valuable insights into MDDF risk factors in Bangladesh, it is important to acknowledge its limitations. First of all, due to data limitations, this study is unable to utilise several important factors that are major contributors to MDD among under-five children. Secondly, the cross-sectional characteristics of the data do not allow us to establish causal relationships. On the other hand, it is also crucial to acknowledge the non-significant findings in our study. For example, while certain factors showed significant associations with inadequate dietary diversity in the bivariate analysis, they may not have retained significance in the multivariate analysis after adjusting for other factors. This suggests that the observed associations might be confounded or influenced by other variables included in the model.

## Conclusion

Based on the findings of this study, it is evident that the rate of MDD among children aged 6–23 months in Bangladesh is low, especially at the district level. Consequently, there is a crucial need for Bangladesh to prioritise efforts in the district areas to enhance the minimum level of dietary diversity among children aged 6–23 months. Additionally, this study highlights that male children have a lower proportion of experiencing MDDF compared to female children. Therefore, it is essential to address the issue of MDD among girls from low socio-economic backgrounds and mothers with limited or no formal education. In conclusion, the insights obtained from this research can serve as valuable guidance for health policymakers in making informed decisions regarding healthcare in district areas of Bangladesh. Our study provides important policy implications for improving dietary diversity among children in Bangladesh. These include addressing gender disparities, enhancing maternal education, targeting poverty reduction, utilising media platforms for education, and strengthening maternal health services. Implementing these policies can contribute to reducing the prevalence of minimal dietary diversity failure and improving the overall nutritional status and well-being of children in Bangladesh.

## References

[1] Saha UR, Chattapadhayay A & Richardus JH. Trends, prevalence and determinants of childhood chronic undernutrition in regional divisions of Bangladesh: evidence from demographic health surveys, 2011 and 2014. PLoS ONE. 2019;14(8):e0220062. 31398208 10.1371/journal.pone.0220062 PMC6688800

[2] Aboagye RG, Seidu AA, Ahinkorah BO, et al. Dietary diversity and undernutrition in children aged 6–23 months in Sub-Saharan Africa. Nutrients. 2021;13(10):3431. 34684435 10.3390/nu13103431 PMC8537414

[3] Borkotoky K, Unisa S & Gupta AK. State-level dietary diversity as a contextual determinant of nutritional status of children in India: a multilevel approach. J Biosoc Sci. 2017;50(1):26-52. 28215213 10.1017/S0021932017000013

[4] Solomon D, Aderaw Z & Tegegne TK. Minimum dietary diversity and associated factors among children aged 6–23 months in Addis Ababa, Ethiopia. Int J Equity Health. 2017;16(1):181-189. 29025434 10.1186/s12939-017-0680-1 PMC5639776

[5] Baek Y & Chitekwe S. Sociodemographic factors associated with inadequate food group consumption and dietary diversity among infants and young children in Nepal. PLoS ONE. 2019;14(3):e0213610. 30856209 10.1371/journal.pone.0213610 PMC6411102

[6] United Nations Children's Fund (UNICEF), World Health Organization & International Bank for Reconstruction and Development/The World Bank. Levels and Trends in Child Malnutrition: Key Findings of the 2021 Edition of the Joint Child Malnutrition Estimates. Geneva: World Health Organization; 2021. 1-32.

[7] Keno S, Bikila H, Shibiru T & Etafa W. Dietary diversity and associated factors among children aged 6 to 23 months in Chelia District, Ethiopia. BMC Pediatr. 2021;21:556. 34895180 10.1186/s12887-021-03040-0 PMC8665635

[8] Beyene M, Worku AG & Wassie MM. Dietary diversity, meal frequency and associated factors among infant and young children in Northwest Ethiopia: a cross-sectional study. BMC Public Health. 2015;15(1007):1-9. 26433689 10.1186/s12889-015-2333-x PMC4592571

[9] Selvaraj K, Stephen T, Priyadharshini SP, Radhakrishnan N & Ashic M. Exploration of dietary diversity and its associated factors among infant and young children in rural Tamil Nadu – a mixed-method study. Indian J Public Health. 2021;65(3):218. 34558481 10.4103/ijph.IJPH_1355_20

[10] Suresh S, Paxton A, Pun BK, et al. Degree of exposure to interventions influences maternal and child dietary practices: evidence from a large-scale multisectoral nutrition program. PLoS ONE. 2019;14(8):e0221260. 31449529 10.1371/journal.pone.0221260 PMC6709950

[11] Ali M, Arif M & Shah AA. Complementary feeding practices and associated factors among children aged 6–23 months in Pakistan. PLoS ONE. 2021;16(2):e0247602. 33630931 10.1371/journal.pone.0247602 PMC7906416

[12] Blackstone S & Sanghvi T. A comparison of minimum dietary diversity in Bangladesh in 2011 and 2014. Mater Child Nutr. 2018;14(4):e12609. 10.1111/mcn.12609 PMC6866105 29663657

[13] Hossain M, Haq I, Zinnia MA, Mila MS & Nayan M. Regional variations of child development index in Bangladesh. Heliyon. 2021;7(5):e07140. 34113735 10.1016/j.heliyon.2021.e07140 PMC8170501

[14] Haq I, Hossain M, Parvin M, Saleheen AAS, Habib M & Chowdhury I-AQ. Gender differences in child nutrition status of Bangladesh: a multinomial modeling approach. J Humanit Appl Soc Sci. 2021;4(5):1-14.

[15] Sheikh N, Akram R, Ali N, et al. Infant and young child feeding practice, dietary diversity, associated predictors, and child health outcomes in Bangladesh. J Child Health Care. 2019;24(2):260-273. 31159554 10.1177/1367493519852486

[16] Ali NB, Tahsina T, Hoque D, et al. Association of food security and other socio-economic factors with dietary diversity and nutritional statuses of children aged 6–59 months in rural Bangladesh. PLoS ONE. 2019;14(8):e0221929. 31465509 10.1371/journal.pone.0221929 PMC6715227

[17] Adubra L, Savy M, Fortin S, et al. The Minimum Dietary Diversity for Women of Reproductive Age (MDD-W) indicator is related to household food insecurity and farm production diversity: evidence from rural Mali. Curr Dev Nutr. 2019;3(3):nzz002. 30899899 10.1093/cdn/nzz002 PMC6423422

[18] Thorne-Lyman AL, Bevis LEM, Kuo H, et al. Season of data collection of child dietary diversity indicators may affect conclusions about longer-term trends in Peru, Senegal, and Nepal. Curr Dev Nutr. 2021;5(8):nzab095. 34466772 10.1093/cdn/nzab095 PMC8397594

[19] Sema A, Belay Y, Solomon Y, et al. Minimum dietary diversity practice and associated factors among children aged 6 to 23 months in Dire Dawa city, Eastern Ethiopia: a community-based cross-sectional study. Glob Pediatr Health. 2021;8:2333794X2199663. 10.1177/2333794X21996630 PMC7905725 33748344

[20] Bedada Damtie S, Benti Tefera T & Tegegne Haile M. Dietary diversity practice and associated factors among children aged 6–23 months in Robe town, Bale zone, Ethiopia. J Nutr Metab. 2020;2020:1-8. 10.1155/2020/9190458 PMC7350076 32685209

[21] Belew AK, Ali BM, Abebe Z & Dachew BA. Dietary diversity and meal frequency among infant and young children: a community based study. Italian J Pediatr. 2017;43(73):1-10. 10.1186/s13052-017-0384-6 PMC5558775 28810887

[22] Paul S, Paul S, Gupta AK & James KS. Maternal education, health care system and child health: evidence from India. Soc Sci Med. 2022;296:114740. 35091129 10.1016/j.socscimed.2022.114740

[23] Vikram K & Vanneman R. Maternal education and the multidimensionality of child health outcomes in India. J Biosoc Sci. 2019;52(1):57-77. 31112112 10.1017/S0021932019000245 PMC7068132

[24] Senarath U, Godakandage SSP, Jayawickrama H, Siriwardena I & Dibley MJ. Determinants of inappropriate complementary feeding practices in young children in Sri Lanka: secondary data analysis of Demographic and Health Survey 2006–2007. Mater Child Nutr. 2011;8:60-77. 10.1111/j.1740-8709.2011.00375.x PMC6860785 22168519

[25] Dangura D & Gebremedhin S. Dietary diversity and associated factors among children 6–23 months of age in Gorche district, southern Ethiopia: cross-sectional study. BMC Pediatr. 2017;17(6):1-7. 28068965 10.1186/s12887-016-0764-x PMC5223415

[26] Kuhnt J & Vollmer S. Antenatal care services and its implications for vital and health outcomes of children: evidence from 193 surveys in 69 low-income and middle-income countries. BMJ Open. 2017;7(11):e017122. 10.1136/bmjopen-2017-017122 PMC5695442 29146636

[27] Torlesse H, Benedict RK, Craig HC & Stoltzfus RJ. The quality of maternal nutrition and infant feeding counselling during antenatal care in South Asia. Mater Child Nutr. 2021;17(3):e13153. 10.1111/mcn.13153 PMC8189234 33554434

[28] ReliefWeb. Rapid Nutrition Assessment in Flood Affected Areas: Sylhet and Sunamganj District - 23–26 August 2022 - Bangladesh | ReliefWeb [Internet]. reliefweb.int. 2022 [cited 2023 Jul 30]. Available from: https://reliefweb.int/report/bangladesh/rapid-nutrition-assessment-flood-affected-areas-sylhet-and-sunamganj-district-23-26-august-2022

